# Notes on the Local Treatment of Certain Diseases of the Skin

**Published:** 1876-10

**Authors:** L. Duncan Bulkley


### NOTES ON THE LOCAL TREATMENT OF CERTAIN DISEASES OF THE SKIN.

BY L. DUNCAN BULKLEY, A. M., M. D.

In a former article* I directed attention to the fact that in the practice of dermatology there was a constant necessity for discrimination in the use of local measures, and that diseases of the skin were to be treated on principles corresponding much to those applying to general medicine, and that while harsh and stimulating remedies may be successful in the hands of those well versed in their use, in many cases, the same treatment indiscriminately employed is fraught with danger here. Some mention was made of the use of baths, especial reference being to full water baths, medicated to suit the case, as will be detailed later on. A word may here be given in reference to sulphur vapor baths, which I constantly see misused. The impression is common that they are of much service in diseases of the skin, and they are ordered for the most diverse states. Sulphur has attained its reputation in this class of disease largely from its almost specific power over scabies, when properly used, but in my experience sulphur baths as ordinarily given have but little effect on the itch, and cannot compare with a thorough use of sulphur ointment. In psoriasis sulphur vapor baths are sometimes of service, but I have now under my care a young lady in whom six sulphur vapor baths, ordered by a previous physician for a very local psoriasis, caused the development of a very general, greatly inflamed eruption, eczematous in nature, covering all of both arms, the neck, thighs, &c., to the great discomfort of the patient. This form of treatment is very seldom applicable to eczema, but where the disease is very chronic, and there are no moist portions, and the skin is found to be not very susceptible to external irritants, it may be recommended with advantage, but must always be prescribed with precaution. I am pretty sure that I have seen more harm done in general from the use of sulphur vapor baths in the hands of others, than I have witnessed good, when I have ordered them.

Having premised this much in general, I will detail briefly the

---

*Archives of Dermatology, April, 1876, p. 212.

local treatment I am in the habit of using in public and private practice in the various diseases affecting the skin, and for conveniencc of writing and reference, I will refer them in alphabetical order. It will be understood that I do not propose to enter the subject of the general treatment of these diseases, nor do I by any means wish to place local treatment before the management of the patient, and the proper use of internal remedies. The object of this article is only to report for the use of the general practitioner, the local measures which may be safely employed, as I have used them conjointly with constitutional treatment. I will not quote authorities, nor even give credit for any of the prescriptions, for it would be a very difficult task to state whence my information was derived in many instances, or whether the practice was original with myself.

I. *Acne.* More cases of acne require, in my judgment, a sooth ing or moderately astringent local treatment than one stimulating or harsh in character. Perhaps the most universally applicable agent is hot water, which most certainly relieves the inflamed masses of very acute acne, and more sluggish elements of indurated and rosaceous acne. The water, as hot as can be borne, is applied with a cloth, holding it to the face for a few moments repeatedly, until every portion of the eruption has been soaked several times. The face is then dried and an astringent lotion is applied; the one I commonly use is as follows: R. potass. Sulphuret., Zinci Sulphat, aa dr. i.; Aquæ Rosæ oz. iv. M. When there is more inflammation, and there is burning and some itching, still more soothing applications are necessary, and this lotion may be applied repeatedly during the day: R. Pulv. Calamin. prep., dr. i.; Zinci Oxidi, dr. i.; Glycerin., dr. ij.; Aquæ Rosæ vel Aquæ Sambucci, oz. iv. M.

When the masses are larger and more obstinate, I find very good results from a wash of Ether and Sulphur, thus: R. Sulph. lot., dr. i.; Etheris Sulph., dr. iv.; Alcoh. oz. iii ss., M. Not unfrequently far more stimulating applications are required in acne, and use is made of potash applications, soft soap, etc. But these are not to be used when the face is inflamed, or when there is much development of new acne elements; when the skin is dry, when comedones are abundant, and when the papules, or pustules are sluggish I use with advantage a wash of caustic potash, from 5 to 15 grains to the ounce of rosewater (far more frequently the former strength and rarely the latter), and this is followed by one of the following ointments, mentioned in the order of increasing strength: R. Zinci Oxidi, dr. i.; Ung. Aquæ Rosæ, oz. i. M.: R. Unguent. Hyd. Precip. Alb., dr. ij.; Ung. Aquæ Rosæ, dr. vj. M.: R. Unguent. Hydrarg, Oxid. Rub., dr. ij.; Ung. Aquæ Rosæ, dr. vj.; M.: R. Unguent. Hydarg. Nitrat., dr. ij.; Unguent. Aquæ Rosæ, dr. vj. M.—These may be further increased in strength as the exigencies of the case demand.

A very important point to be borne in mind in prescribing for acne is that if a mercurial and a sulphur application are made conjointly, or immediately following each other, chemical change takes place, and the skin is very unpleasantly darkened, often causing much alarm; patients should therefore be cautioned to allow at least 24 hours to elapse, with repeated bathing in hot water, between the use of any preparations containing sulphur and those with mercury or lead.

When the production of inflammatory papules has about ceased, I find advantage from the use of the German green soap, sapo viridis, which the patient rubs on the skin pretty firmly with flannel and warm water at night. If any of these applica-cations dry the skin too much, I find much benefit from the use of sweet cream from milk, (a saucer of good milk standing over night will produce enough cream for a day's use), or the Vaseline answers very well, or ordinary cold cream, Ung. Aquæ Rosæ.

The comedones, or clogged subaceous glands, are best emptied by pressure upon them with the end of a small tube, with an aperture of about 1-16 of an inch, or a new watch-key; the orifice is placed over the little black speck, and firm pressure is made perpendicular to the surface, when, in most cases, the worm-like mass will rise partly or completely from its gland, and may be readily removed. The masses in indurate acne should be opened with a lancet inserted perpendicularly, as soon as pus is discovered; it is also best to open all suppurating glands as early as practicable.

II. *Alopecia.* Alopecia is such an indefinite term, and baldness may result from so many causes, that it is different to lay down any definite line of local treatment. Where it is the result of a seborrhœa, in which case the dandruff is a prominent feature, the treatment of this is necessary to the cure of the former. Locally the following ointment has proved serviceable in my hands in removing the scaliness and restoring the hair: R. Tinct. Cantharid. dr. i.; Ung. Hydrarg. Nitrat., dr. ij. Ung Aquæ Ros. dr. vj. Ol. Amygdal, Amaræ. gtt. ij. M.

Where the loss of hair is the result of ringworm, and occurs in spots, with a dirty, slightly scaly surface, and nibbled off hairs, the parasite disease must be removed before the hair can be restored. In the fall of hair due to syphilis, it is best not to attempt any local treatment until its advance has been arrested by internal treatment, as the applications made will be pretty certain to have the immediate effect of increasing the shedding of the hair; after the hairs have ceased to fall, the following wash may be used, though I question if the hair is not reproduced quite as quickly without any local treatment: R. Tinct. Catnarid, dr. ij.; Tinct. Capici, dr. iv.: Olei Ricini, oz. ss.; Aquæ Cologniensis, ad. oz. iv. M.

The same application is a valuable hair stimulant in the loss of hair after febrile diseases, and in simple debility, the proportion of the cantharides and capsicum may be increased until slight tinging and redness follows a pretty firm application, with a bit of flannel. Quinine is also a good hair tonic, and may be used in the proportion of fifteen or twenty grains to the ounce, with half a drachm of tincture of nux vomica, and equal proportions of bay rum and rose water.

II. *Bromiadrosis.* Offensive sweating of the hands, feet, axillæ, etc., is generally associated with or dependent upon excessive sweating or hyperidrosis, and the two may therefore be considered together. I have had good results from bathing the parts once or twice a day with tincture of belladonna, or it may be rubbed in, care being taken not to produce too great constitution effect by it. About the best local treatment for the excessive sweating of the feet is the Unguentum diachyli of the German Pharmacopœia, applied twice a day, and worn continuously next to the skin. I have long used it and found it generally successful, but the belladonna is cleanlier and perhaps equally efficacious. Salicylic acid also gives good results either used in solution, a drachm or two to the ounce, with a little borax, or in powder, thus; R. Acidi Salicylici, dr. iv.; Zinci Carbonat. precip., dr. iv.; Magnes. ustæ, oz. iv., Pulvis Lycopodii, oz. i ss. M.

Cleanliness is, of course, all important, and the dipping the feet into very hot water before the application of the remedies will facilitate the cure, and the addition of a moderate proportion of tannin in the water (from two to four drachms to the pint) will further assist.—*Archives of Dermeatology.*

